# The Clinical Characteristics of New-Onset Epilepsy in the Elderly and Risk Factors for Treatment Outcomes of Antiseizure Medications

**DOI:** 10.3389/fneur.2022.819889

**Published:** 2022-02-22

**Authors:** Jing Qi, Xiao Liu, Na Xu, Qun Wang

**Affiliations:** ^1^Department of Neurology, Beijing Tiantan Hospital, Capital Medical University, Beijing, China; ^2^National Center for Clinical Medicine of Neurological Diseases, Beijing, China; ^3^Collaborative Innovation Center for Brain Disorders, Beijing Institute of Brain Disorders, Capital Medical University, Beijing, China

**Keywords:** antiseizure medications, treatment outcomes, elderly, new-onset epilepsy, risk factor

## Abstract

**Objective:**

To describe the clinical characteristics of elderly patients with new-onset epilepsy in a Class A tertiary comprehensive hospital in north China and evaluate the treatment outcomes of antiseizure medications (ASMs). This study focuses on investigating the factors affecting the treatment outcomes, guiding the drug treatment, and judging the prognosis of elderly epilepsy patients.

**Methods:**

We included patients aged 60 years or older at the time of their first seizure between January 2014 and August 2020. Demographic characteristics, effects of ASM, and the proportion of 1-year and long-term seizure freedom were reported. The univariate analysis and binary logistic regression were used to identify factors potentially influencing treatment outcomes.

**Results:**

A total of 326 patients (median age 65 years, 67.2% men) were included. Moreover, 185 (56.7%) patients who received the first ASM monotherapy achieved 1 year of seizure freedom in the early stage. Compared with structural etiology, unknown etiology was associated with a higher likelihood of early seizure freedom (odds ratio [*OR*] = 0.545; *p* < 0.05). Conversely, comorbid intracranial malignant tumors, taking carbamazepine (CBZ), and sodium valproate (VPA) were associated with a lower likelihood of seizure freedom (*OR* = 3.527 vs. 6.550 vs. 8.829; *p* < 0.05). At long-term follow-up, 263 (80.6%) patients achieved seizure freedom, with 79.8% on monotherapy.

**Conclusions:**

Elderly patients with new-onset epilepsy responded well to the initial ASMs treatment. Patients with intracranial malignant tumors and prescribed VPA and CBZ were less likely to achieve early seizure freedom, while those with unknown etiology had higher probabilities of achieving early seizure freedom than those with structural etiology.

## Introduction

With the aging of the population, the incidence of elderly epilepsy is significantly higher than that of any other age group (1), and the economic burden on individuals and the healthcare system continues to increase. The clinical characteristics and drug selection of elderly patients with epilepsy are different from those of younger patients. The incidence of status epilepticus is higher, almost two times that of young people (2), so it is necessary to choose reasonable antiseizure medication (ASM) as soon as possible. Due to changes in pharmacokinetics and pharmacodynamics, increased systemic comorbidities, complex drug interactions, and poor medication compliance, elderly people are more prone to drug-related adverse events. As various physiological conditions of the elderly change with age, many factors can affect the outcomes of treatment.

Data published in the past few years have identified several risk factors related to the prognosis of adult epilepsy. Factors, such as the number of seizures before treatment, complex partial seizures, the etiology of abnormal brain structure, longer initial treatment time, depression, and abnormal brain imaging were related to the adverse treatment outcomes of epilepsy (3–7), but most of the studies were conducted in the whole or adult population. So far, few studies have explored the influencing factors of ASM on the treatment outcomes of elderly patients with new-onset epilepsy, especially in the Chinese population. Therefore, this paper described the clinical characteristics of elderly patients with new-onset epilepsy in our center, evaluated the treatment outcomes of different ASMs, and investigated factors that affect the outcomes of the treatment, aiming to find more reasonable management regimens for elderly patients with new-onset epilepsy.

## Materials and Methods

### Study Design and Participants

This retrospective study was conducted at Beijing Tiantan Hospital, Capital Medical University, a Class A tertiary comprehensive hospital in northern China. We searched all electronic medical records to include newly diagnosed elderly epilepsy patients who were admitted to the epilepsy outpatient department of our hospital or transferred to the epilepsy wards between January 2014 and August 2020 and received ASM as monotherapy. Treatment regimens were altered as necessary depending on the efficacy and tolerability. Generally speaking, the original ASM was replaced if it caused intolerable adverse reactions at low doses or if it did not improve the seizure control. If the first well-tolerated ASM significantly improved the number of seizures but failed to provide complete control, combination therapy was considered. The inclusion criteria were as follows: (1) a diagnosis of epilepsy fulfilling the 2014 International League Against Epilepsy (ILAE) diagnostic criteria (8); and (2) age at first seizure onset ≥ 60 years. The exclusion criteria were as follows: (1) acute symptomatic seizures that were secondary to substances (such as alcohol abuse) or withdrawal or seizures due to acute illness; (2) missing cases, lost to follow-up, death within 1 year of medication use or not receiving ASM treatment; and (3) poor treatment outcomes due to unsatisfied medication-taking compliance or inadequate dosage of the first ASM. All subjects were followed for at least 1 year to record seizures and medications.

### Variables and Definitions

The electronic medical records were reviewed to collect the patient's gender, age of first seizure, type of seizure, etiology, comorbidity, seizure frequency before treatment, concomitant medications, brain imaging examination, and interictal electroencephalogram (EEG) before treatment. We recorded the use of ASM in detail, including the time from the first onset to receive ASM treatment, type and number of ASM, dosage, efficacy, and reasons for withdrawal (adverse reactions, poor therapeutic effect, long-term seizure freedom, or death). Seizure types were classified as focal seizures, focal to bilateral generalized tonic-clonic seizures, and tonic-clonic seizures of unknown onset or unclassified based on semiology and interictal EEG findings (9). The etiology of epilepsy was documented in the case records, including structural, metabolic, infectious, immune, and unknown. Moreover, we collected the comorbidities of patients, including coronary heart disease, atrial fibrillation, and valvular heart disease. Psychiatric disorders included anxiety, depression, and schizophrenia. A history of mild to moderate traumatic brain injury (TBI) was recorded (10). The frequency of seizure was classified as daily (1 or more times per day), persistent (< 1 seizure per day but at least 1 seizure in the past 6 months), rare (< 1 seizure per 6 months), and undefined (seizure frequency could not be specified according to recent seizures) (11). The use of aspirin and statins, which have been shown to reduce the frequency of seizures and have neuroprotective effects, was also collected (12–15). Neuroimaging findings were divided into normal, non-epileptogenic abnormalities, and epileptogenic abnormalities. Three patients received only CT scans due to metal implants, while the rest received 3.0 T MRI scans.

Based on the outcomes of treatment, all patients were divided into a seizure-free group, that is, patients achieved seizure freedom 12 months after initial ASM treatment and a failed treatment group. It was defined as seizures even after taking a full dose of ASM for 1 year or changing ASM or polytherapy combinations due to poor efficacy or side effects. All patients were followed until 30 August 2021 or death.

### Statistical Analysis

The above data of patients were statistically analyzed by SPSS (IBM SPSS Statistics 26.0, NY, USA). We used descriptive statistics to assess frequencies and distributions. A chi-squared test or Fisher's exact test was used for inter-group comparison of categorical variables. Variables with *p* < 0.2 in the univariate analysis were included in binary logistic regression analysis to determine independent influencing factors. We screened variables with backward stepwise (likelihood ratio). A value of *p* < 0.05 was considered significant.

## Results

### Demographics and Clinical Characteristics

**Figure 1:**
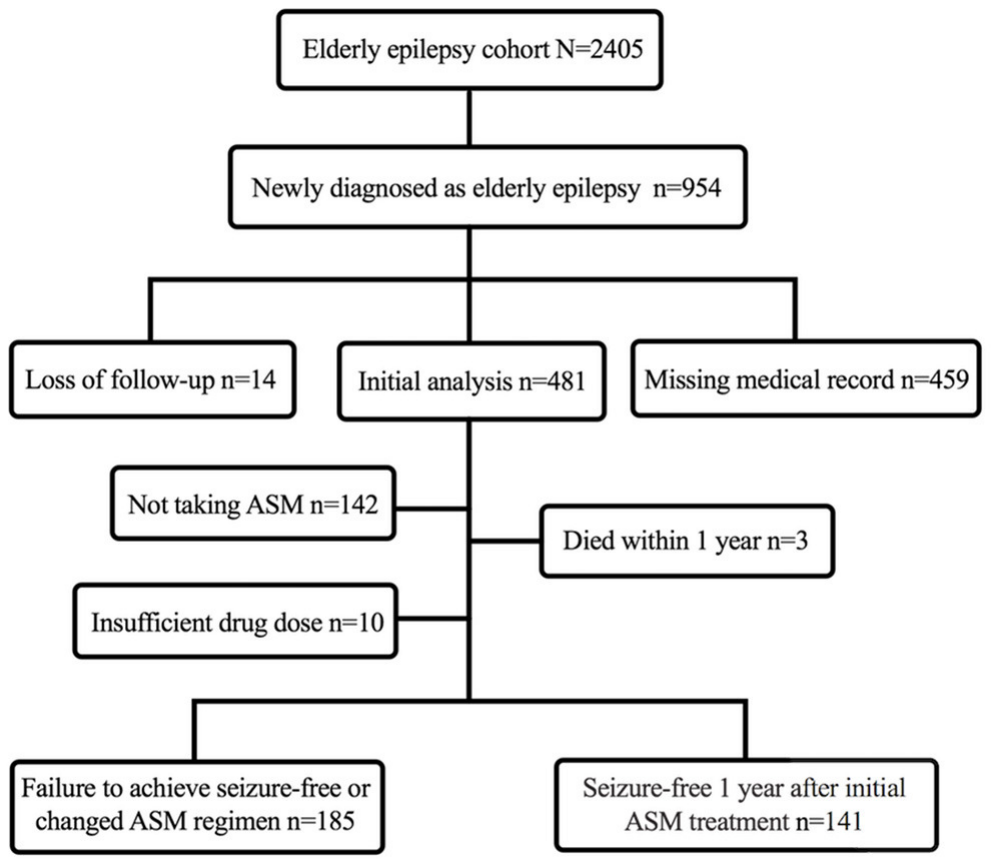
Flowchart of constructing a cohort of new-onset epilepsy in the elderly.

In this retrospective study, a total of 326 elderly patients with new-onset epilepsy were included (Figure 1). The median age of onset was 65 years (range 60–90 years) and 219 (67.2%) were men (Table 1). Structural etiology accounted for the highest proportion, followed by unknown etiology. The most common identifiable etiology was cerebrovascular disease (*n* = 97, 30.0%), followed by intracranial tumors (*n* = 29, 8.9%), autoimmune encephalitis (AE) (*n* = 25, 7.7%), and TBI (*n* = 22, 6.7%). Focal seizures (*n* = 299, 91.7%) were more common than tonic-clonic seizures of unknown onset (*n* = 10, 3.1%), with the majority of patients presenting as secondary generalized tonic-clonic seizures (*n* = 186, 62.2%). Of the patients initially treated with ASM monotherapy, 185 (56.7%) patients achieved seizure freedom and 141 (43.3%) patients failed in treatment.

**Table 1:** Demographic data of elderly patients with new-onset epilepsy, *N* (%).

	**Seizure-free group (*n* = 185)**	**Failed treatment group (*n* = 141)**	**Total (*n* = 326)**	***P-*value^a^**
Age of onset (years)				0.307^b^
60–69	123 (66.5)	101 (71.6)	224 (68.7)	
70–79	43 (23.2)	32 (22.7)	75 (23.0)	
≥80	19 (10.3)	8 (5.7)	27 (8.3)	
Gender				0.761^b^
Male	123 (66.5)	96 (68.1)	219 (67.2)	
Female	62 (33.5)	45 (31.9)	107 (32.8)	
Seizure type				0.121^b^
Focal seizures	62 (33.5)	51 (36.2)	113 (34.7)	
Focal Seizures with secondary generalization	107 (57.8)	79 (56.0)	186 (57.1)	
Tonic-clonic seizures of unknown onset	3 (1.6)	7 (5.0)	10 (3.1)	
Unclassified Seizures	13 (7.0)	4 (2.8)	17 (5.2)	
Etiology of epilepsy				0.003^c^
Structural	96 (51.9)	85 (60.3)	181 (55.5)	
Immune	8 (4.3)	17 (12.1)	25 (7.7)	
Infectious	2 (1.1)	3 (2.1)	5 (1.5)	
Metabolic	3 (1.6)	2 (1.4)	5 (1.5)	
Unknown	76 (41.1)	34 (24.1)	110 (33.7)	
Time from first seizure to initiation of the first ASM (months)				0.056^b^
<3	91 (49.2)	90 (63.8)	181 (55.5)	
3–6	21 (11.4)	13 (9.2)	34 (10.4)	
6–12	24 (13.0)	10 (7.1)	34 (10.4)	
>12	49 (26.5)	28 (19.9)	77 (23.6)	
Comorbidity				
Stroke	66 (35.7)	48 (34.0)	114 (35.0)	0.759^b^
Hypertension	81 (43.8)	64 (45.4)	145 (44.5)	0.772^b^
Diabetes	34 (18.4)	26 (18.4)	60 (18.4)	0.989^b^
Cardiovascular disease	29 (15.7)	19 (13.5)	48 (14.7)	0.579^b^
Intracrainial benign tumors	11 (5.9)	11 (7.8)	22 (6.7)	0.508^b^
Intracrainial malignant tumors	4 (2.2)	10 (7.1)	14 (4.3)	0.030^b^
Central nervous system infection	2 (1.1)	3 (2.1)	5 (1.5)	0.656^b^
Traumatic brain injury	9 (4.9)	14 (9.9)	23 (7.1)	0.077^b^
Psychological disorders	19 (10.3)	13 (9.2)	32 (9.8)	0.752^b^
Neurodegenerative diseases	30 (16.2)	17 (12.1)	47 (14.4)	0.289^b^
MRI or CT scan at entry				0.218^b^
Epileptogenic abnormalities	91 (49.2)	83 (58.9)	174 (53.4)	
Non-epileptogenic abnormalities	39 (21.1)	25 (17.7)	64 (19.6)	
Normal	55 (29.7)	33 (23.4)	88 (27.0)	
Interictal EEG				0.335^b^
Epileptiform	91 (49.2)	81 (57.4)	172 (52.8)	
Slowing	64 (34.6)	41 (29.1)	105 (32.2)	
Normal	30 (16.2)	19 (13.5)	49 (15.0)	
Seizure frequency at onset				0.252^b^
Daily	37 (20.0)	35 (24.8)	72 (22.1)	
Persistent	129 (69.7)	98 (69.5)	227 (69.6)	
Rare	14 (7.6)	4 (2.8)	18 (5.5)	
Unclassified	5 (2.7)	4 (2.8)	9 (2.8)	
Concomitant drugs				
Aspirin	50 (27.0)	30 (21.3)	80 (24.5)	0.232^b^
Statins	63 (34.1)	40 (28.4)	103 (31.6)	0.274^b^
Type of first ASM				<0.001^c^
LEV	85 (45.9)	29 (20.6)	114 (35.0)	
OXC	65 (35.1)	37 (26.2)	102 (31.3)	
VPA	17 (9.2)	54 (38.3)	71 (21.8)	
CBZ	6 (3.2)	14 (9.9)	20 (6.1)	
LTG	11 (5.9)	6 (4.3)	17 (5.2)	
PB	0 (0)	1 (0.7)	1 (0.3)	
TPM	1 (0.7)	0 (0)	1 (0.3)	

a *The value of p between the seizure-free group and failed treatment group groups*.

b *Pearson's chi-square, two-sided*.

c *Fisher's exact test, two-sided*.

*LEV, levetiracetam; OXC, oxcarbazepine; VPA, sodium valproate; CBZ, carbamazepine; LTG, lamotrigine; PB, phenobarbital; TPM, topiramate; CT, computed tomography; MRI, magnetic resonance imaging; EEG, electroencephalogram; ASM, antiseizure medication*.

### Univariate Analysis

The important variables related to the treatment outcomes of initial monotherapy included the etiology, comorbid intracranial malignant tumors, and the types of ASM. The proportion of metabolic and unknown etiology in seizure-free patients was higher than that of patients with treatment failure (1.6 and 41.1% vs. 1.4 and 24.1%, respectively, *p* < 0.05). In contrast, the proportion of patients with structural, infectious, and immune etiologies was lower, and the proportion of comorbid intracranial malignant tumors was lower (2.2 vs. 7.1%, *p* < 0.05). The number of patients receiving levetiracetam (LEV) (*n* = 114, 35%) was the largest, followed by oxcarbazepine (OXC) (*n* = 102, 31.3%), VPA (*n* = 72, 21.8%), CBZ (*n* = 20, 6.1%), and lamotrigine (LTG) (*n* = 17, 5.2%). The dosage of different ASMs is shown in Table 2. Among 114 elderly patients who received LEV, 85 (45.9%) patients achieved seizure freedom and 29 patients (20.6%) failed treatment. In addition, 65 (35.1%) patients treated with OXC achieved early 1-year seizure freedom, while 26.2% (*n* = 37) of patients failed treatment. Furthermore, 54 patients (38.3%) who received VPA and 14 patients (9.9%) who received CBZ had unsatisfactory treatment outcomes. Among the patients receiving VPA, 8 (14.8%) patients changed ASM regimens due to intolerable side effects, including thrombocytopenia, dizziness, and tremor. Among the patients treated with CBZ, 7 (50%) patients changed treatment regimens due to side effects, mainly skin rash and leukopenia. Table 3 lists the reasons for the failure of treatment.

**Table 2:** Outcomes of antiseizure medication (ASM) monotherapy and their doses within 1 year.

**Monotherapy**	**Seizure-free group**			**Failed treatment group**		
	**n (%)**	**Median dose (mg)**	**IQR**	**n (%)**	**Median dose (mg)**	**IQR**
LEV (*n* = 114)	85 (74.5)	1,000	1,000	29 (25.4)	1,000	1,000–1,500
OXC (*n* = 102)	65 (63.7)	600	600–900	37 (36.3)	600	600–900
VPA (*n* = 71)	17 (23.9)	1,000	1,000	54 (76.1)	1,000	1,000
CBZ (*n* = 20)	6 (30.0)	400	250–400	14 (70.0)	400	200–400
LTG (*n* = 17)	11 (64.7)	100	75–100	6 (35.3)	100	100

*LEV, levetiracetam; OXC, oxcarbazepine; VPA, sodium valproate; CBZ, carbamazepine; LTG, lamotrigine; IQR, interquartile range*.

**Table 3:** Reasons and proportion of treatment failure with the first ASMs.

**ASM**	**n**	**Lack of efficacy (%)**	**Side effects (%)**
LEV	29	26 (89.7%)	3 (10.3%)
OXC	37	22 (59.5%)	15 (40.5%)
VPA	54	46 (85.2%)	8 (14.8%)
CBZ	14	7 (50.0%)	7 (50,0%)
LTG	6	4 (66.7%)	2 (33.3%)
PB	1	1 (100.0%)	0 (0%)

*ASM, antiseizure medication; LEV, levetiracetam; OXC, oxcarbazepine; VPA, sodium valproate; CBZ, carbamazepine; LTG, lamotrigine; PB, phenobarbital*.

### Multivariate Analysis

**Figure 2:**
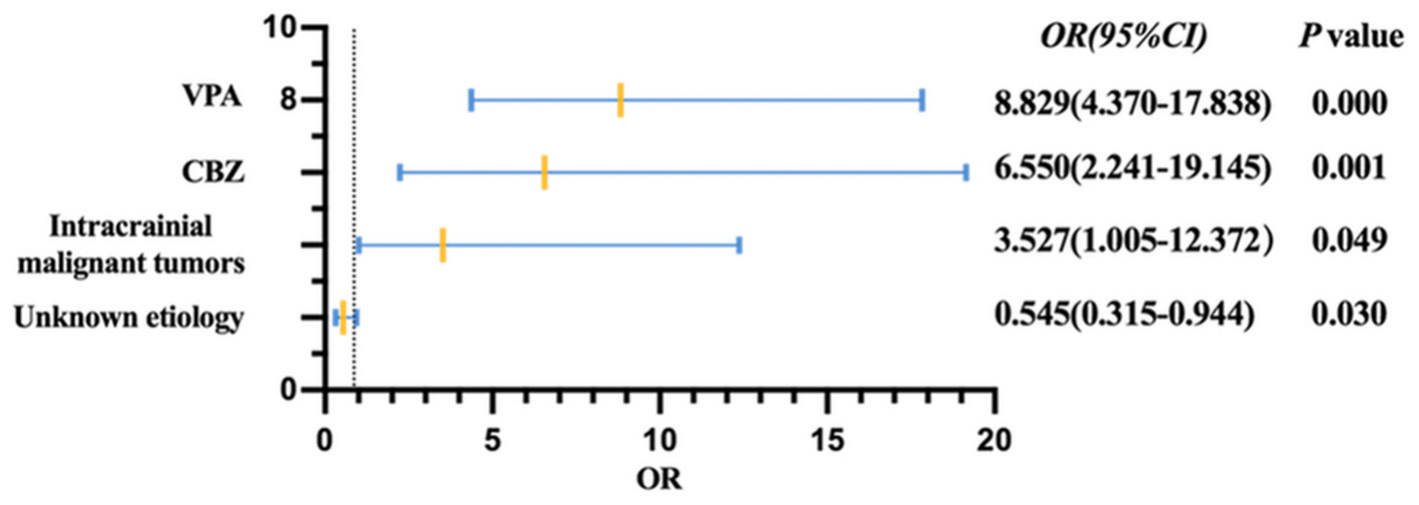
Forest plot of multivariate logistic regression with backward stepwise. The reference categories were not complicated with intracranial malignant tumors, not treated with CBZ and VPA. VPA, sodium valproate; CBZ, carbamazepine; OR, odds ratio; CI, confidence interval.

After excluding the interference of the time from the first onset to treatment, the history of TBI, and the type of seizure, there were three risk factors associated with lower likelihood of early seizure freedom in elderly patients, including intracranial malignant tumor (odds ratio [*OR*] = 3.527, 95% *CI* = 1.005–12.372, *p* = 0.049), who received CBZ (*OR* = 6.550, 95% *CI* = 2.241–19.145, *p* = 0.001), and VPA (*OR* = 8.829, 95% *CI* = 4.370–17.838, *p* < 0.001). Compared with the structural etiology, unknown etiology was associated with higher likelihood of seizure freedom (*OR* = 0.545, 95% *CI* = 0.315–0.944, *p* = 0.030) (Figure 2).

### Long-Term Epilepsy Treatment Outcomes

All patients were followed for 12–84 months, with a median follow-up time of 42 months (interquartile range [IQR] = 27–47 months). At the last follow-up, 263 (80.6%) patients were seizure-free by taking different ASM regimens. Of 229 patients who received only one type of ASM, 210 (91.7%) patients were seizure-free (Table 4). When combining two ASMs, LEV combined with OXC was the most common (*n* = 27, 38.0%), followed by VPA combined with OXC (*n* = 18, 25.4%), and VPA combined with LEV (*n* = 26, 36.6%). Comparing the three groups, LEV combined with VPA (seizure-free rate was 45.2%) or OXC (40.5%) predicted better treatment outcomes (χ^2^ = 7.215, *p* = 0.027).

**Table 4:** One-year seizure-free rate of successive ASM regimens at last follow-up.

**Type of ASM**	**Number of patients taking the ASM regimen**	**Patients achieving seizure-free**			
		**n**	**% of patients who took the ASM regimen**	**% of 263 patients achieving seizure freedom**	**% of total 326 patients**
One	229	210	91.7	79.8	64.4
Two	88	50	56.8	19.0	15.3
Three	7	2	28.6	0.8	0.6
Four	2	1	50.0	0.4	0.3
Total	326	263		100.0	80.6

*ASM, antiseizure medication*.

## Discussion

This study investigated elderly patients with new-onset epilepsy in the past 7 years and assessed the comprehensive variables available at the time of diagnosis and identified factors that affect the outcomes of ASM monotherapy in elderly patients with epilepsy. In this study, two main conclusions were as follows: (1) elderly patients with newly diagnosed epilepsy responded well to initial ASMs, and patients with unknown etiology predicted favorable ASM treatment outcomes. (2) Combined intracranial malignant tumors and initial ASM therapy with VPA and CBZ were associated with a lower likelihood of early seizure freedom.

According to the new 2017 ILAE classification, the etiology of epilepsy includes structural, metabolic, immune, infectious, and unknown. However, up to 25–50% of elderly patients have no identifiable etiology. In our study, patients with unknown etiology were more likely to achieve seizure freedom than patients with structural etiology. This phenomenon was confirmed in the adult cohort (5). The current popular hypothesis is that late-onset unexplained epilepsy is related to occult cerebrovascular disease (16). In Sarkis et al.'s recent study on elderly patients with epilepsy of unknown etiology, patients were sensitive to ASM. At the last follow-up, 92% of patients did not have seizures. The incidence of smoking, antihypertensive treatment, and sleep apnea was higher (17). In addition, a study on the risk factors of midlife and the development of epilepsy revealed similar findings. The author suggested that hypertension, smoking, and diabetes mellitus were significantly associated with the development of epilepsy (18). In our study, 40.9 and 14.5% of the patients with unknown etiology had hypertension and diabetes mellitus. The mechanism of these risk factors leading to seizures is not clear but may include the induction of neuroinflammation, the destruction of neurovascular units, the destruction of the blood-brain barrier, and oxidative stress. In Sarkis et al.'s study, about one-third of patients were found to have high levels of periventricular hyper signal (Fazekas scores 2–3), while a subset of patients had medial temporal lobe atrophy, suggesting underlying Alzheimer's disease pathology (17). The treatment outcomes may be related to the absence of significant structural damage and that this favorable treatment response reflects the lower epileptogenic potential of the underlying lesions encountered in this age group, as well as a lower genetic predisposition to refractory epilepsy (19). Therefore, doctors should evaluate vascular risk factors and investigate the presence of hippocampal atrophy in patients with late-onset unknown epilepsy at initial diagnosis and follow-up the changes of brain imaging for a long time. In recent years, antibody-mediated epilepsy has emerged as one of the etiologies of epilepsy in the elderly, with data suggesting that AE may explain at least 20% of adult epilepsy of unknown etiology (20). In our study, some patients with unknown etiology had varying degrees of memory loss, but the patients refused to do the antibody detection related to autoimmune encephalitis in cerebrospinal fluid and serum. Consequently, the exaggerated proportion of patients with unknown etiology caused by AE could not be completely ruled out. In the future, the relationship between vascular risk factors and epilepsy of unknown etiology in the elderly and whether the intervention of these risk factors will change the prognosis of patients will be further explored.

In our study, elderly patients with intracranial malignant tumors (mainly brain metastases and high-grade gliomas) were associated with poor epilepsy control. Similarly, in the study of Hersi, the seizure-free rate of adult patients with intracranial malignant tumors after receiving the first ASM treatment was 3.4 and 7.6% of patients still suffered from seizures. High-grade gliomas and brain metastases are the most common malignant tumors causing seizures (21). It is difficult to control seizures in these patients with ASM alone. The association between intracranial malignant tumors and unsatisfactory treatment outcomes may be explained by the following hypotheses. First, the target of ASM binding may change in tumors and peritumoral tissues. Second, multi-drug transporters that transport various ASMs (LEV and LTG) are overexpressed in brain tumors. The upregulation of multi-drug transporters identified in epileptogenic brain tissues may inhibit the entry of ASMs into epileptogenic brain tissues. Overexpression of multidrug transporters in brain tumors has been reported, which may be the reason for drug refractoriness observed in patients with a brain tumor. However, this hypothesis does not seem to hold in metastatic brain tumors. Because the vascular system of metastatic brain tumors shows the characteristics of primary tumor vessels, which change the characteristics of the blood-brain barrier. In other words, drug resistance through enhanced expression of multi-drug transporters is less of a problem in metastatic brain tumors. Finally, the neurobiological factors that cause the severity of the disease contribute to the development of drug resistance (22).

Sodium valproate has long been one of the preferred ASM for gliomas with epilepsy, partly due to its inherent antitumor effect (23). Redjal et al. found that VPA was dose-dependent with the improvement of the survival rate of patients with glioblastoma. Unexpectedly, in grade II and III gliomas, VPA was linked to histological progression and a decrease in progression-free survival (24). Studies evaluated the efficacy of ASM in patients with grade II-IV gliomas and found that compared with topiramate (TPM), VPA, CBZ, and OXC, LEV and phenytoin sodium (PHT) seemed to be the most effective monotherapy and LEV was well-tolerated. Considering that the seizure-free rate of VPA was relatively low, the author did not support VPA as the first-line single-drug regimen, but VPA could be a good choice for second-line ASM combined with LEV to treat patients with uncontrolled epilepsy (25). Data on the efficacy of ASM in elderly patients with brain metastases are scarce. LEV and VPA are probably the most appropriate drugs. Studies have shown that total tumor resection was effective in controlling seizures and provided better seizure control at follow-up than subtotal resection (26). In addition to the positive effects of surgical resection, a growing body of data suggested that radiation and chemotherapy played a role in reducing the frequency of seizures in patients with gliomas (27, 28). Therefore, every patient with an epileptic malignant brain tumor should be treated first by resection, radiotherapy, and chemotherapy to eliminate the tumor and epileptic focus. Only when this treatment plan cannot effectively control epileptic seizures, ASM treatment should be strengthened (25).

In this study, we provided that patients treated with CBZ and VPA were associated with adverse treatment outcomes. About 85.2% of patients taking VPA switched to or combined with a second ASM due to poor drug efficacy, while 50% of patients taking CBZ switched to other ASM due to intolerable side effects. Although ILAE proposed CBZ and VPA as initial monotherapy for newly diagnosed partial epilepsy in the elderly, there was only grade D evidence given that first-generation ASMs had more adverse reactions. A *post-hoc* analysis of a randomized controlled trial compared LEV, CBZ, and VPA in elderly patients with epilepsy. The results showed that the discontinuation time of LEV was the longest, and the discontinuation rate of LEV was the lowest at 12 months. But the time of first seizure recurrence was similar among the three groups. Adverse events were reported by 76.2, 67.3, and 82.5% of patients for LEV, VPA, and CBZ, respectively (29). In Werhahn et al.'s study, 359 elderly patients with focal epilepsy were included. The median daily doses of CBZ, LTG, and LEV were 380, 95, and 950 mg/day, respectively. The retention rate of LEV was significantly higher than that of CBZ (61.5 vs. 45.8%). There was no difference in seizure-free rate among the groups. Patients who discontinued CBZ due to adverse events were two times as compared with LEV (32.2 vs. 17.2%) (30). In line with most studies, we do not recommend CBZ or VPA as the first choice for elderly patients with epilepsy due to side effects, while LEV is a safer drug with a similar or higher seizure-free rate. There was no statistically significant difference in other variables between groups except that the number of seizures before treatment showed a tendency of poor treatment effect (*p* = 0.056). A network meta-analysis estimated the comparative efficacy and safety of ASMs [CBZ, LTG, LEV, lacosamide (LCM), gabapentin, and phenytoin] in the elderly with new-onset epilepsy and showed that there was no significant difference in efficacy across ASMs treatments. LCM, LTG, and LEV ranked best in achieving seizure freedom with the highest probability. CBZ showed a poor tolerability profile, resulting in higher withdrawal rates (31). A large amount of data support that second-generation ASMs (e.g., LTG and LEV) are as effective as traditional ASMs and well-tolerated, so they remain the preferred medications for the elderly. In addition, the third-generation ASMs (e.g., LCM, brivaracetam, perampanel, and eslicarbazepine acetate) have a favorable pharmacokinetic profile, better tolerability, and fewer drug-drug interactions, which are undoubtedly the more appropriate choice for elderly patients. But there are limited data available in clinical trials on the third-generation ASMs in elderly patients (32).

The study has several limitations, notably that it was a retrospective study based on medical records, which prevented us from accurately estimating the effect of certain clinical variables on treatment outcomes or drawing conclusions about which ASM was more effective. Most patients receiving monotherapy were treated with LEV, OXC, or VPA, so the outcomes data were more reflective of these specific ASMs. A certain selection bias was also inevitable. Owing to some patients only having the reports of brain imaging or electronic medical records, we cannot obtain accurate and detailed imaging information to further categorize neuroimaging abnormalities. Although there are limitations, to our knowledge, few studies have explored the factors affecting the results of initial monotherapy in elderly patients with new-onset epilepsy. As an authoritative epilepsy diagnosis and treatment center in China, the patients we admitted were from all over the country, increasing the diversity of individuals. In addition, we included epilepsy patients with AE into the analysis to make the etiology distribution more reasonable and more reliable.

This study highlights that an initial monotherapy in elderly patients with newly diagnosed epilepsy often has favorable outcomes. We found several factors affecting the early treatment outcome, among which, comorbid intracranial malignant tumor and the treatment with CBZ and VPA predicted poor treatment outcomes, while the patients whose etiology could not be determined through comprehensive history collection and necessary auxiliary examination might predict relatively favorable treatment outcomes. LEV is undoubtedly a safe and effective choice for the elderly, but its mental and emotional side effects should be paid attention to.

## Data Availability Statement

The raw data supporting the conclusions of this article will be made available by the authors, without undue reservation.

## Ethics Statement

The studies involving human participants were reviewed and approved by the Ethics Committee of the Beijing Tiantan Hospital that was affiliated with the Capital Medical University of the People's Republic of China. The patients/participants provided their written informed consent to participate in this study. Written informed consent was obtained from the individual(s) for the publication of any potentially identifiable images or data included in this article.

## Author Contributions

QW: conceptualized, designed, and supervised this study. JQ: collected, screened the data, performed the statistical analysis, and drafted the manuscript. XL, NX, and QW: critically revised the important content in the manuscript. All authors read and approved the final manuscript.

## Funding

The study was financially supported by the National Key R&D Program of China grant (2017YFC1307500 to QW), the Capital Health Research and Development of Special grants (2016-1-2011 and 2020-1-2013 to QW), the Beijing-Tianjin-Hebei Cooperative Basic Research Program (H2018206435 to QW), and the Beijing Natural Science Foundation (Z200024 to QW).

## Conflict of Interest

The authors declare that the research was conducted in the absence of any commercial or financial relationships that could be construed as a potential conflict of interest.

## Publisher's Note

All claims expressed in this article are solely those of the authors and do not necessarily represent those of their affiliated organizations, or those of the publisher, the editors and the reviewers. Any product that may be evaluated in this article, or claim that may be made by its manufacturer, is not guaranteed or endorsed by the publisher.
